# Spermatocytic tumor associated with metastases: report of a rare case and literature review

**DOI:** 10.3389/fphar.2025.1636142

**Published:** 2025-08-04

**Authors:** Jianbai Chen, Xiaorong Mou, Zhiming Zhang, Wei Zhang, Zhiyong Nie, Xiaoping Gao, Yanyao Gao, Jianxin Qiu

**Affiliations:** Tangdu Hospital, Fourth Military Medical University, Xi’an, China

**Keywords:** spermatocytic tumor, metastasis, chemotherapy, sarcomatous differentiation, disease progression

## Abstract

Spermatocytic tumor (ST) is an exceedingly rare testicular neoplasm with non-specific clinical presentations, requiring histopathological confirmation for diagnosis. Radical orchiectomy remains the cornerstone of treatment, achieving favorable outcomes in most cases. However, early metastasis observed in some patients emphasizes the necessity of comprehensive systemic staging at diagnosis and rigorous postoperative surveillance. Current evidence is largely derived from case reports, limiting robust clinical guidance. Herein we present a case of pure testicular ST with synchronous multiple pulmonary metastases managed at Tangdu Hospital. Despite undergoing radical orchiectomy followed by chemotherapy, the patient exhibited disease progression. Additionally, we performed a literature review of published ST cases to summarize its clinicopathological features, treatment paradigms, and prognostic patterns. This study highlights the challenges in managing advanced ST and underscores the need for standardized therapeutic protocols.

## Introduction

Testicular tumors, while uncommon, represents about 5% of all urogenital tract tumors. Recent years have shown an increase in its incidence, particularly in developed nations (Park et al., 2018). In the United States, an estimated 9,760 new cases were diagnosed in 2024, while in the People’s Republic of China, around 3,500 new cases were reported in 2022 (Siegel et al., 2024; Zheng et al., 2024). Spermatocytic tumor, a rare type of testicular tumor, has limited research and understanding due to its uncommon nature. According to the 2016 World Health Organization (WHO) classification system for testicular tumors, ST is classified as a germ cell tumor distinct from primary germ cell neoplasms. The first reported case of spermatocytic seminoma dates back to 1946, when Dr. Masson first discovered and described it.

Due to the rarity of testicular ST, the biological and clinicopathological characteristics remain largely elusive. Current knowledge of this disease is mainly based on small series and case reports. Currently, no consensus has been reached in standard treatment strategy for patients with ST apart from radical orchiectomy. Furthermore, the underlying mechanisms responsible for the sarcomatoid transformation observed in a subset of testicular ST cases and the early distant metastasis observed in some others remain elusive. In this study, we reported a case of metastatic testicular ST at our tertiary center. In addition, to achieve better understanding of the disease, we performed a literature review with an attempt to describe the clinical and pathological characteristics and treatment strategy of ST of the testis.

## Case description

**FIGURE 1:**
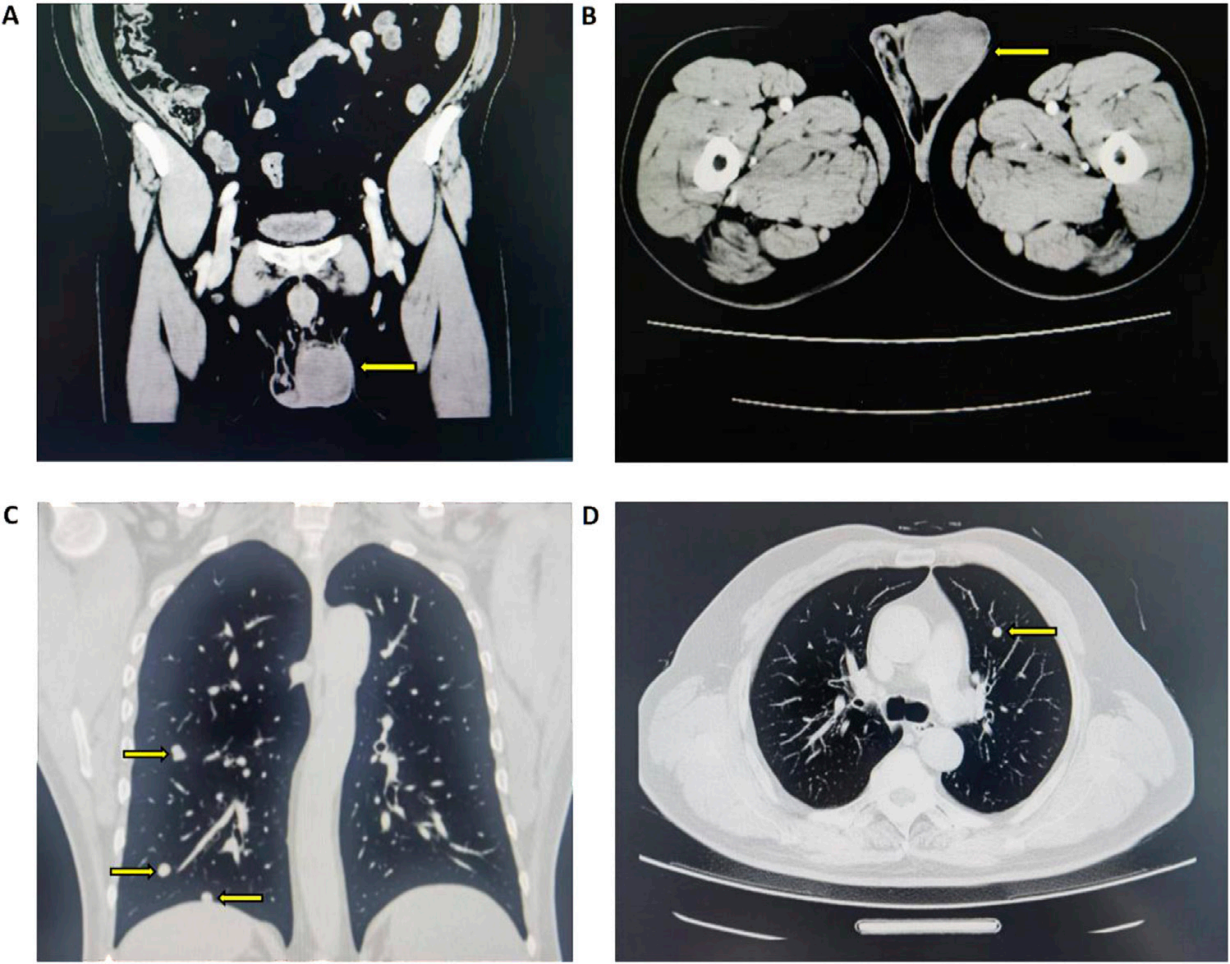
CT imaging findings of left testicular neoplasm and bilateral pulmonary nodules **(A)** Coronal view of left testicular mass-like slightly hyperdense focus with mild enhancement **(B)** Axial view of corresponding left testicular lesion **(C)** Coronal view of bilateral pulmonary nodules **(D)** Axial view of bilateral pulmonary nodule.

A 53-year-old male patient was admitted to Tangdu Hospital due to a swollen left testis with dull pain for 1 month. He denied a history of cryptorchidism and symptoms like frequent urination, urgency, dysuria, hematuria, or urination difficulties. No fever, severe testicular pain, or breast development was reported. Physical examination revealed an enlarged left testis with a firm, irregular surface. There was no tenderness, and the testis had normal mobility. During laboratory investigations, tumor markers such as AFP, HCG, and LDH were within the normal ranges. Ultrasound indicated hypoechoic nodules in the left testis, while the whole body CT imaging revealed a mass-like slightly hyperdense focus within the left testis, which demonstrated mild enhancement on contrast-enhanced scans—findings consistent with a left testicular neoplasm. The chest CT further disclosed multiple bilateral pulmonary nodules, likely representing metastatic tumor, as shown in Figure 1. To clarify the characteristics of the pulmonary lesion, the patient underwent an ultrasound-guided needle biopsy of the right lung mass. The biopsy findings indicated a germ cell tumor, with the testis being considered as the potential origin of the tumor. The clinical diagnosis was left testicular tumor with multiple metastases in both lungs, requiring surgery. On 22 April 2024, the patient underwent a radical resection of the left testicular tumor. Notably, as there was no evidence of lymph node enlargement detected in the preoperative imaging examination of the patient, no lymph node dissection was performed during the surgical procedure.

**FIGURE 2:**
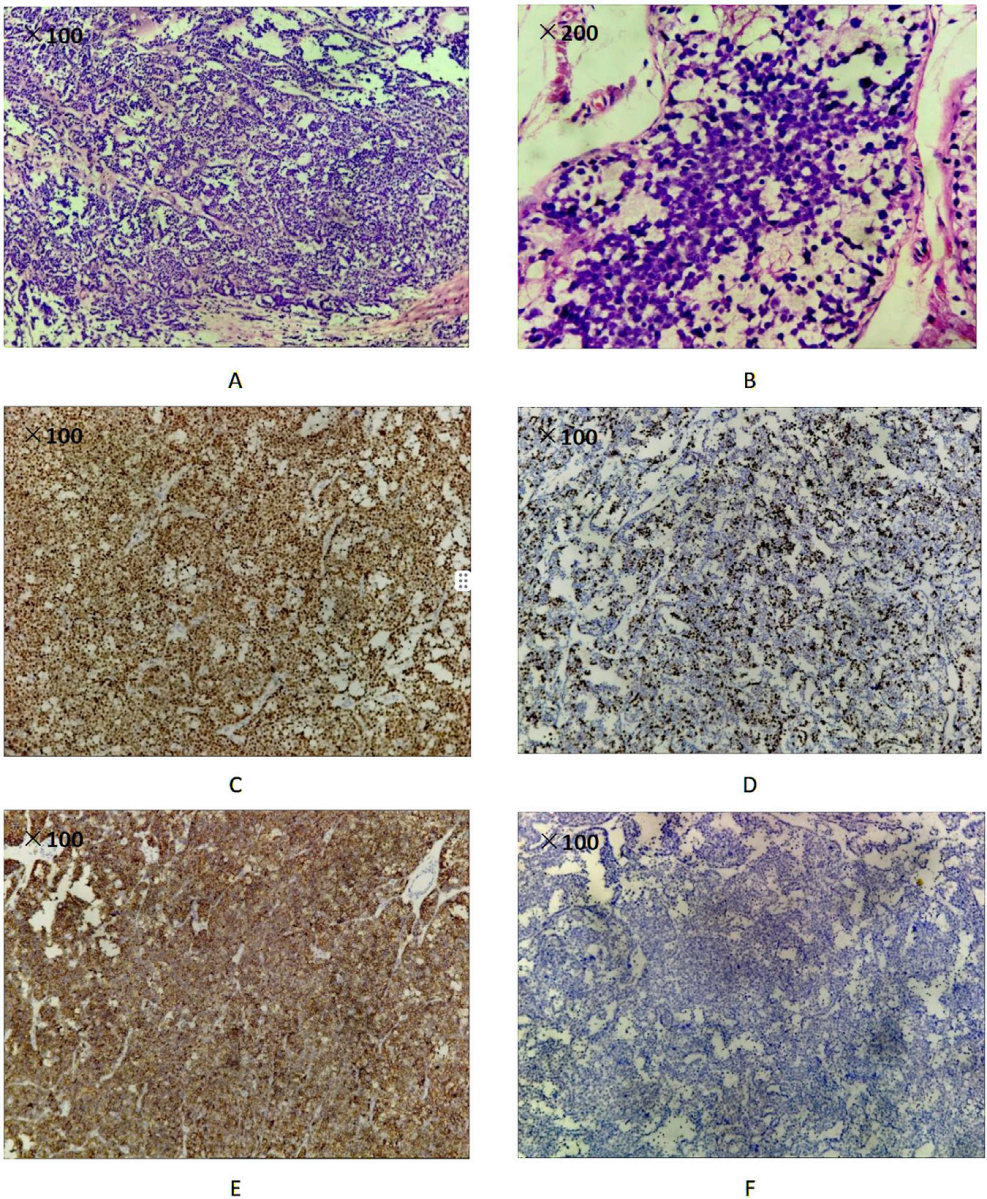
Pathology images and immunological staining outcomes of the testicular tumor **(A)** H&E magnification ×100. Microscopic features of spermatocitic tumor **(B)** H&E magnification ×200. Microscopic features of spermatocitic tumor **(C)** Spermatocytic tumor cells show cytoplasmic positivity with SALL4 (100x) **(D)** Ki‐67 proliferation index was 60% (100x) **(E)** Spermatocytic tumor cells show strong membrane staining for CD117 (100x) **(F)** Spermatocytic tumor cells show negative for OCT3/4 (100x).

The postoperative pathological examination revealed a radical resection specimen of the left testis measuring 5.0 cm × 4.0 cm x 3.2 cm. Upon inspection of the testicular cut surface, a grayish-white tumor mass measuring 4.0 cm × 3.0 cm x 3.0 cm was observed. The tumor had a fleshy appearance with a soft texture and clear boundary. Microscopically, hematoxylin and eosin staining showed nodular proliferation of tumor cells within a fibrous and edematous stroma. Lymphocyte infiltration was absent. The tumor cells displayed round morphology in three sizes - large, medium, and small. Surrounding cells did not show aberrant changes, indicating an *in situ* germ cell tumor. The final pathological stage was T1N0M1. Immunohistochemical analysis revealed positive markers for SALL4, CD117, and CD56 in the tumor cells. The proliferation marker Ki-67 showed up to 60% expression, as illustrated in Figure 2.

Following the patient’s diagnosis of multiple metastases in both lungs, chemotherapy consisting of etoposide, cisplatin, and bleomycin was initiated 2 weeks post-surgery. The BEP regimen included cisplatin administered at 20 mg/m^2^ on days 1–5 via intravenous infusion, etoposide at 100 mg/m^2^ on days 1–5 via intravenous infusion, and bleomycin at 30 mg on days 2, 9, and 16 via intramuscular injection, with a 21-day interval between cycles. By 20 June 2024, the patient had completed two cycles of BEP chemotherapy without experiencing any significant adverse effects. During the third cycle of the BEP chemotherapy regimen, grade IV bone marrow suppression was observed, which resolved following symptomatic management with human granulocyte colony-stimulating factor (G-CSF). Subsequent to completion of the third chemotherapy cycle, the patient elected to discontinue further BEP-based treatment. No additional chemotherapy or surgical interventions were pursued thereafter. Follow-up chest CT imaging performed on 12 November 2024, demonstrated stable bilateral pulmonary lesions without evidence of new metastatic lesions, consistent with a classification of stable disease (SD). Subsequent whole-body CT surveillance identified multiple bilateral retroperitoneal lymphadenopathies (1 right-sided and 2 left-sided enlarged nodes), with the largest measuring 11 mm in maximum diameter. No interval changes were detected in the pulmonary lesions. These findings suggested disease progression, with tumor staging reaffirmed as T1N1M1. Despite clinical recommendations, the patient declined further therapeutic options due to personal reasons. Long-term monitoring will be continued to assess disease trajectory.

## Discussion

Testicular germ cell tumors (GCTs) are classified into type I, II, and III based on their developmental origin, histological features, molecular alterations, and age distribution (Oosterhuis and Looijenga, 2019). Among these, type I (prepubertal) and type II (postpubertal) GCTs exhibit overlapping histologic subtypes but can be distinguished by the presence or absence of germ cell neoplasia *in situ* (GCNIS) (Berney et al., 2016). In contrast, type III GCTs, previously considered a subtype of seminoma, have now been recognized as a distinct tumor entity, termed ST, owing to their unique clinical features, biological behavior, and molecular characteristics (Honecker et al., 2018). ST is thought to arise from neoplastic transformation of more mature germ cell progenitors and, importantly, are not associated with GCNIS (Oosterhuis and Looijenga, 2019; Giannoulatou et al., 2017). ST is a rare germ cell tumor that specifically arises in the testis, representing only 1% of all testicular GCTs. On average, individuals are diagnosed with testicular spermatocytic seminoma at the age of 52, which is approximately 15 years older than seminoma and 25 years older than embryonal carcinoma. The distribution between left and right testicular sides is almost equal (51% vs. 44%), with a small percentage (5%) of patients experiencing bilateral onset (HU et al., 2019). Tumor sizes can vary between 14 mm and 250 mm, with an average diameter of 57 mm. Previous research has highlighted cryptorchidism as an independent risk factor for testicular GCTs, but this theory does not apply to patients with ST (Moller et al., 1998). Notably, only Stevens and Hu have reported cases of patients with testicular spermatocytic seminoma who had a history of cryptorchidism (Hu et al., 2019; Stevens et al., 1993). Additionally, although rare, there have been previous report documenting the coexistence of leydig cell tumor and spermatocytic tumor within the same testis (ANDO and NAITO, 2024).

In order to gain a deeper understanding of ST, a literature search was conducted using databases such as PubMed, Cochrane, Embase, CNKI, and WANFANG to identify English and Chinese articles on the subject. Search terms included “Testis” and “Spermatocytic tumor”, “Spermatocytic seminoma”, or “Spermatocytic”. The search deadline was set for December 2024. Additionally, reference lists of relevant articles were reviewed for additional cases. Data extracted included information on first author, publication year, patient demographics, clinical characteristics, radiographic and pathological findings, and treatment approaches. Summary of characteristics and prognosis of patients with metastatic and histologically variant spermatocytic tumors. We made a summary of characteristics and prognosis of patients with metastatic and histologically variable spermatocytic tumors in previous cases, specifically shown in Tables 1–3. The detailed information for the Chinese case data presented in Table 3 were listed in Supplementary Table S1.

**TABLE 1:** Reported cases of conventional spermatocytic tumors associated with metastases.

Number	Author (year)	Age (y)	Side	Size (mm)	Pathology factors	Initial staging	Metastases and location	Treatment	Follow up (months after diagnosis)
1	Our case	53	Left	40	ST	Ⅲa	Lung	OE + CT	9-PD
2	Choi et al. (2017)	77	Left	NA	ST	Ⅲc	Brain	OE + RT	PD
3	Steiner et al. (2006)	26	Right	70	ST	I	RPLN	OE + RPLND + CT (BEP)	36-NED
4	Caty et al. (1997)	73	Right	70	ST	II	RPLN	OE + RT	6-DOD
5	Böhm et al. (1993)	39	Left	60	ST	I	RPLN	OE + RPLND + CT (BEP)	LTFU
6	Matoška et al. (1988)	49^a^	Left	NA	ST	I	RPLN	OE + RPLND + CT + RT	25-DOD
7	Schoborg et al. (1980)	61	Right	NA	ST	II	Multiple LN (Retroperitoneal, Axillary, Supraclavicular) + Cheek + Thigh	OE + CT + RT	9-DOD

^a^ This patient died of the complications of chemotherapy.

ST = spermatocytic tumor; RPLN = retroperitoneal lymph node; LN = lymph node; OE = orchiectomy; CT = chemotherapy; RT = radiotherapy; RPLND = retroperitoneal lymph node dissection; NA = not available; SD = stable disease; PD = progressive disease; NED = no evidence of disease; DOD = died of disease; LTFU = lost to follow up.

**TABLE 2:** Reported cases of histologically variant spermatocytic tumors.

Number	Author (year)	Age (y)	Side	Size (mm)	Pathology factors	Initial staging	Metastases and location	Treatment	Follow up (months after diagnosis)
1	Sarabia et al. (2024)	27	Both	NA	AS	I	None	OE + CT	LTFU
2	Ando and Naito (2024)	84^a^	Right	15	Leydig cell tumor	I	None	None	6-DOD
3	Rabade et al. (2022)	60	Right	100	AS	I	None	OE + RT	110-NED
4		59	Left	120	AS	I	None	OE	72-NED
5		52	Right	NA	AS	I	None	OE	LTFU
6		80	Right	NA	SAUS	I	None	OE + RT + CT	10-DOD
7		55	Right	100	SARS	NA	NA	OE + CT	LTFU
8	Dias et al. (2023)	46	Right	180	SARS	Ⅲc	Lung + Brain + Mediastinal LN	OE + CT (VIP)	10-DOD
9	Chennoufi et al. (2021)	66	Left	170	SAUS	Ⅲc	Lung + Bone + RPLN	OE + CT (VIP)	7-DOD
10	Pandey et al. (2018)	60	Right	180	SARS	NA	None	OE	2-NED
11	Hu et al. (2019)	52	NA	95	SAUS	NA	NA	NA	LTFU
12		39	NA	45	SAUS	NA	NA	NA	LTFU
13	Stueck et al. (2017)	52	Left	95	SAUS	I	None	OE	41-NED
14	Jeong et al. (2015)	52	Left	90	SARS	Ⅲc	Left ureter + Neck LN + RPLN	OE + CT (VIP + TIP) + RT	4-NED
15	Mikuz et al. (2014)	40	Right	NA	AS	I	Lung + Liver + Kidney + RPLN	OE + CT (BEP + IEP) + Resection of retroperitoneal mass	41-PD
16	Gentile et al. (2014)	63	Both	200 (L) 100 (R)	AS	I	None	OE	36-NED
17	Wetherell et al. (2013)	29	Right	60	SAUS	I	None	OE	LTFU
18	Narang et al. (2012)	38	Right	70	SARS	I	None	OE + RT + CT (VAC)	2-NED
19	Lombardi et al. (2011)	46	Left	80	AS	I	None	OE + CT	LTFU
20	Trivedi et al. (2011)	43	Left	180	SAUS	I	Lung	OE + CT (BEP)	10-DOD
21	Menon et al. (2009)	55	Right	150	SARS	I	None	OE	LTFU
22	Robinson et al. (2007)	44	NA	170	SARS	Ⅲc	Bone + Brain	OE + CT (VIP)	5-DOD
23	Dundr et al. (2007)	56	Left	100	AS	I	Lung	OE + CT	8-PD
24	Chelly et al. (2006)	50	Right	140	SARS	Ⅲc	Liver	OE + CT	3-DOD
25	Albores-Saavedra et al. (1996)	38	Right	55	AS	I	None	OE + RT	38-NED
26		33	Right	100	AS	I	None	OE + RT	27-NED
27		43	Left	80	AS	I	None	OE + RT	4-NED
28		42	Left	25	AS	I	None	OE + RT	3-NED
29	Sabater-Marco et al. (1994)	42	Left	80	SARS	I	None	OE + CT	10-NED
30	Eble (1994)	39b	NA	NA	SAUS	NA	NA	OE	LTFU
31		68	NA	NA	SAUS	NA	NA	OE	9-DOD
32		34	NA	NA	SAUS	NA	NA	OE	LTFU
33		68	NA	NA	SAUS	NA	NA	OE	11-DOD
34	Matoska and Talerman (1990)	51	Right	180	SARS	Ⅲc	Lung + Liver + Bone + RPLN	OE	2-DOD
35	True et al. (1988)	55	Left	50	SAUS	I	Retroperitoneal mass	OE	24-NED
36		66	Right	170	SAUS	I	None	OE + RT	96-DOD
37		56^b^	Left	90	SARS	I	Lung	OE + RPLND + CT (BEP)	15-DOD
38		40	Right	75	SAUS	I	None	OE	9-NED
39		60	NA	250	SARS	Ⅲc	Lung + Multiple LN + Heart + Thyroid + Peritoneum + Small and large bowel	None	1-DOD
40	Floyd et al. (1988)	42	Left	95	SAUS	I	Lung + RPLN	OE + CT	12-DOD

^a^ This patient died of heart failure.

^b^ The case data was derived from the article by Eble in 2004.

AS = anaplastic spermatocytic; SAUS = spermatocytic associated with undiferentiated sarcoma; SARS = spermatocytic associated with rhabdomyosarcoma; RPLN = retroperitoneal lymph node; LN = lymph node; OE = orchiectomy; CT = chemotherapy; RT = radiotherapy; RPLND = retroperitoneal lymph node dissection; VIP, etoposide + ifosfamide + cisplatin, TIP = paclitaxel + ifosfamide + cisplatin, BEP = bleomycin + etoposide + cisplatin, IEP = ifosfamide + etoposide + cisplatin, VAC = vincristine + actinomycin + cyclophosphamide, NA = not available; SD = stable disease; PD = progressive disease; NED = no evidence of disease; DOD = died of disease; LTFU = lost to follow up.

**TABLE 3:** Reported cases of spermatocytic tumors in China.

Number	Author (year)	Age (y)	Side	Size (mm)	Pathology factors	Initial staging	Metastases and location	Treatment	Follow up (months after diagnosis)
1	Zhang et al., 2023	57	Right	35	ST	I	None	OE	9-NED
2		52	Right	50	SARS	Ⅲc	Lung + Bone	OE + CT (BEP) + RT + Resection of bone metastases	34-PD
3	Yan et al., 2022	34	Right	55	ST	I	None	OE	LTFU
4	Yang et al., 2020	39	Right	25	ST	I	None	OE	NED
5		61	Left	140	ST	I	None	OE	NED
6	Peng et al., 2015	18^a^	Left	160	SARS	Ⅲc	Lung + Bone + RPLN	OE	DOD
7	Tian et al., 2012	29	Right	35	ST	I	None	OE	12-NED
8		45	Left	30	ST	I	None	OE	31-NED
9		53	Right	55	ST	I	None	OE	72-NED
10		66	Right	80	ST	I	None	OE	11-NED
11		71	Right	46	ST	I	None	OE	19-NED
12	Zhang et al., 2012	47	Both	150 (L) 15 (R)	ST	I	None	OE	LTFU
13	Shao et al., 2012	48	Both	75 (L) NA (R)	ST	I	None	OE	10-NED
14	Wang et al., 2011	40	Left	45	ST	I	None	OE	LTFU
15	Gao et al., 2009	45	NA	120	SARS	I	None	OE	LTFU
16	Jia et al., 2004	45	Left	45	SARS	I	None	OE	LTFU
17	Du et al., 2003	71	Both	30 (L) 30 (R)	ST	I	None	OE	LTFU
18	Li et al., 2002	30	Left	NA	ST	I	None	OE	4-NED
19	Zhang et al., 1998	38	Right	30	ST	I	None	OE	LTFU
20	Yan et al., 1995	46	Right	110	ST	I	None	OE	LTFU

^a^ This patient gave up on further treatment for financial reasons.

ST = spermatocytic tumor; SARS = spermatocytic associated with rhabdomyosarcoma; RPLN = retroperitoneal lymph node; LN = lymph node; OE = orchiectomy; CT = chemotherapy; RT = radiotherapy; BEP = bleomycin + etoposide + cisplatin, NA = not available; SD = stable disease; PD = progressive disease; NED = no evidence of disease; DOD = died of disease; LTFU = lost to follow up.

The clinical presentation of ST usually comprises the occurrence of a painless mass accompanied by a continuously enlarged testis, and the tumor tends to be relatively large, averaging between 5 and 7 cm in size. Routine tumor markers like AFP, HCG and LDH are usually within normal ranges, and hormone levels remain stable. Ultrasonography is a noninvasive and preferred method for detecting testicular masses early. The ultrasound findings often show masses with varying echogenicity and strong blood flow signals on Doppler imaging. However, some cases may be complicated by testicular inflammation, leading to potential misdiagnosis. In addition to ultrasound, CT scans offer a more detailed view of the tumor, surrounding structures, and any potential lymph node involvement. While imaging techniques are helpful, they cannot determine the histological type of the tumor, highlighting the importance of pathological examination for definitive diagnosis.

ST typically exhibit a lobulated and multinodular appearance, often with cystic changes, focal necrosis, and potential epididymal invasion. When observed under low magnification, seminomas display a diffuse or multinodular arrangement, with abundant loose fibrous stroma between nodules and significant edema and serous exudate in focal areas. The concentration of fluid within cancer nests can sometimes create a pseudoglandular appearance (Mikuz et al., 2014). The key diagnostic characteristics of ST include the presence of three distinct polymorphous cell populations. Some sarcomatous changes can occur in ST, typically manifest as undifferentiated sarcoma or rhabdomyosarcoma, characterized by spindle cell or pleomorphic morphology (Gupta et al., 2024).

Immunohistochemistry serves as a pivotal tool in the diagnosis of ST. Notably, many commonly encountered embryonic germ cell tumor markers, including OCT3/4, PLAP, alpha-fetoprotein, human chorionic gonadotropin-B, CD30, and transcriptional activator 2 years, exhibited negative expression patterns in ST (Gupta et al., 2024). Conversely, proteins typically expressed in spermatogonia, such as SALL4, MAGED4, CD117, SYT, SAGE 1, Dmrt 1, and OCT2, were predominantly positively expressed in ST (Gupta et al., 2024). Additionally, immunohistochemical characteristics of certain cancer/testis (CT) antigens, including NUT, GAGE 7, and NY-ESO-1, have been documented in ST, providing further insights into its biological profile (Dias et al., 2023). Previous case reports have identified a small subset of patients with unique immunohistochemical findings. For instance, Dias and Jeong observed abnormal LDH levels, reaching twice the normal value (Dias et al., 2023; Jeong et al., 2015). Gentile and Robinson also noted abnormal LDH levels, with both PLAP and CD117 positivity (Gentile et al., 2014; Robinson et al., 2007). It is noteworthy that patients from the Dias and Robinson studies passed away shortly after disease detection, indicating LDH levels may serve as a prognostic indicator for poor disease outcomes. In our case, the tumor exhibited positivity for CD117 and SALL4 while demonstrating negativity for other germ cell tumor markers. These findings were consonant with the immunohistochemical profile of ST. Concurrently, immunohistochemical analysis serves as a crucial tool in the identification and classification of sarcomatous elements. Notably, the components of rhabdomyosarcoma exhibited a strong positive reaction for desmin and a moderate positive response for myoglobin, the undifferentiated spindle cell component demonstrated a positive immunoreactivity solely for vimentin (Dias et al., 2023). Additionally, Increased expression of p53 were found in some anaplastic cases (Gupta et al., 2024).

The study by Eble highlighted that due to the slow-growing nature of ST, a subset of patients experience a considerable delay in seeking medical treatment after detecting the lesion (Eble, 1994). Approximately one-third of patients wait up to 12 months or more before seeking medical attention, with a small percentage waiting up to 5 years or longer (Eble, 1994). The primary treatment for ST is radical orchiectomy, typically without the need for chemotherapy or radiotherapy. While most patients have a favorable prognosis, a minority may develop metastases, commonly in the retroperitoneal lymph nodes, although metastases to the lung, bone, liver, and brain have also been documented. Research by Grogg et al. found that around 7% of patients diagnosed with localized disease initially experienced postoperative metastases (Grogg et al., 2019). Following a median follow-up of 5.5 months (ranging from 2 to 21 months), patients with aggressive histology, such as sarcoma or anaplastic subtype, were more prone to developing metastatic disease (Grogg et al., 2019). Although clear predictors of tumor metastasis are lacking, previous reports have mentioned vascular invasion in the specimen as a potential indicator (Dias et al., 2023).

Metastatic cases are typically managed with a combination of radiotherapy and chemotherapy, although the efficacy of this approach remains uncertain due to the limited number of cases. In the Musa study, the patient received combined adjuvant chemotherapy with cisplatin, vinblastine, and bleomycin but died of septic shock (Musa et al., 1998). In Steiner’s study, the patient underwent 2 cycles of carboplatin after radical orchiectomy but recurred after 10 months, leading to RPLND with BEP (bleomycin, etoposide and cisplatin) chemotherapy. After 3 years, no disease recurrence was observed (Steiner et al., 2006). However, in the Dias study, the patient passed away due to complications 3 months after 4 cycles of VIP (etoposide, cisplatin and ifosfamide) chemotherapy (Dias et al., 2023). In Zhang’s study, a patient with pulmonary and vertebral metastases received 3 cycles of BEP chemotherapy after radical orchiectomy. Though bone metastases showed no significant changes, pulmonary metastases decreased. Remarkably, this patient survived for 40 months. Chemotherapy has achieved good results in some patients, however not all patients can achieve satisfactory results. Some experts argue that the disease may not respond well to chemotherapy, suggesting that a treatment regimen involving both chemotherapy and radiotherapy is more rational, potentially leading to complete response (Jeong et al., 2015; Grogg et al., 2019).

Molecularly, ST is typically aneuploid, with a genome ranging from near-diploid to near-tetraploid. Giannoulatou et al. reported that relative gains of chromosome 9 are almost universally observed in ST, with DMRT1 (located at 9p21.3-pter) identified as a likely driver gene implicated in oncogenesis (Giannoulatou et al., 2017). Furthermore, Gupta et al. conducted genomic analysis of 25 cases of ST and revealed two distinct genomic subgroups, one characterized by global ploidy shifts without recurrent mutations, and the other by a diploid genome harboring RAS/RAF hotspot mutations (Gupta et al., 2024). Notably, while the relative gain of chromosome 9 represents a common characteristic shared by both subgroups, consistent with previous findings, biological progression in ST was significantly associated with relative gains of chromosome 12p and mutations in the TP53 gene. The presence of 12p gains, a hallmark feature of GCNIS-related germ cell tumors, suggests that certain ST may share biological characteristics with GCNIS-derived germ cell tumors rather than non-GCNIS-derived ST. Notably, in cases exhibiting sarcomatoid transformation, TP53 mutations appear to serve as a critical driver of the transition to sarcomatoid histology, which may provide a biological basis for elucidating the mechanisms underlying the aggressive progression of ST (Gupta et al., 2024).

In our study, although the disease was detected at an early stage with localized lesions, absence of vascular/lymphatic invasion, and no evidence of retroperitoneal lymph node metastasis, leading to our decision to omit lymph node dissection during surgery, disease progression occurred 9 months postoperatively despite administration of three cycles of BEP chemotherapy. This suggests that more aggressive therapeutic approaches should be considered for early-stage testicular tumors with distant metastasis. Prophylactic lymph node dissection may be warranted even in the absence of preoperative evidence of nodal involvement, combined with intensified adjuvant therapy. These findings require further validation through additional clinical investigations to establish optimal management strategies for this patient subgroup.

As ST of the testis is rare, the existing literature mainly consists of case reports that focus on exceptional and uncommon clinical cases, which are more likely to be published. These tumors have a higher rate of metastatic disease, particularly in patients with sarcoma or anaplastic subtypes, leading to potential publication bias. The lack of detailed information on tumor stage in previous reports hinders our ability to explore factors influencing tumor recurrence and progression. Limited data are available on the outcomes of patients undergoing chemotherapy and radiotherapy following radical orchiectomy, with patients experiencing metastasis often having a poor prognosis despite these treatments.

This study, the first to include case information from China, addresses gaps in previous retrospective studies and represents one of the largest statistical cohorts of ST to date. Compared to conventional testicular GCTs, ST may exhibit resistance to chemotherapy, especially in cases where patients have sarcoma or anaplastic subtypes, contributing to their unfavorable prognosis post-metastasis. Currently, the treatment of ST relies heavily on expert consensus. We encourage more experts and clinicians to share their treatment experiences with ST, espicially special types and metastatic diseases. Furthermore, prospective studies on ST are eagerly anticipated in the future.

## Data Availability

The original contributions presented in the study are included in the article/Supplementary Material, further inquiries can be directed to the corresponding authors.
