# Supplementary material for: Spermatocytic tumor associated with metastases: report of a rare case and literature review
Source: Front Pharmacol. 2025 Aug 4;16:1636142. doi: 10.3389/fphar.2025.1636142 (PMC12358719; doi:10.3389/fphar.2025.1636142)
Supplement: Supplementary file 1 [file Table1.docx]

| Number | Author (year) | Title | Journal Name | First Author | All authors (in order) | Information of the corresponding author | Volume | Issue | Page number | DOI |
| --- | --- | --- | --- | --- | --- | --- | --- | --- | --- | --- |
| 1 | Zhang Zhongyun, Sun Zhongquan, Sheng Lu, Wang Dongya, Wu Jianhong, Zhang Zhengwang et al., 2023 | Testicular spermatocytic tumor: a report of two cases and literature review | Chinese Journal of Andrology | Zhang Zhongyun | Zhang Zhongyun, Sun Zhongquan, Sheng Lu, Wang Dongya, Wu Jianhong, Zhang Zhengwang, Chai Qiliang and Qian Weiqing | Department of Urology, Affiliated to Fudan University, Shanghai 20040, China | 37 | 03 | 86-89 | 10.3969/j.issn.1008-0848.2023.03.015 |
| 2 | Yan Xiang, Yuan Hongmei, Wen Jiayu, Jian Shunhai, 2022 | Ultrasonic misdiagnosis of spermatocytic seminoma of testis complicated with orchitis: Case report | Chinese Journal of Medical Imaging Technology | Yan Xiang | Yan Xiang, Yuan Hongmei, Wen Jiayu and Jian Shunhai | Department of Ultrasound, Affiliated Hospital of North Sichuan Medical College, China | 38 | 06 | 960-960 | 10.13929/j.issn.1003-3289.2022.06.044 |
| 3 | Yang Shanshan, Wang Xuedi, Zhao Min, Wang Ting, Fu Yao, 2020 | Clinicopathological analysis and literature review of 2 cases of spermatocytic seminoma | Journal of Clinical and Pathological Research | Yang Shanshan | Yang Shanshan, Wang Xuedi, Zhao Min, Wang Ting and Fu Yao | Department of Pathology, Affiliated Drum Tower Hospital, Nanjing University Medical School, Nanjing 210008, China | 40 | 06 | 1612-1617 | 10.3978/j.issn.2095-6959.2020.06.046 |
| 4 | Peng Yeping, Feng Zhenhua, Qiu Guangjin, 2015 | Testicular spermatocytic seminoma with sarcomatous component: A case report | Journal of Modern Urology | Peng Yeping | Peng Yeping, Feng Zhenhua and Qiu Guangjin | Department of Urology, Gaozhou People's Hospital, Guangdong Province, Gaozhou 525200, China | 20 | 07 | 528-528 | 10.3969/j.issn.1009-8291.2015.7.024 |
| 5 | Tian Baoling, Gao Aifeng, Xu Can, Chang Xiaoying, Xu Zhenqun, Su Qinghua et al., 2012 | Clinicopathologic analysis of spermatocytic seminoma | Chinese Journal of Pathology | Tian Baoling | Tian Baoling, Gao Aifeng, Xu Can, Chang Xiaoying, Xu Zhenqun, Su Qinghua and Yang Xianghong | Department of Pathology, Sheng ]ing Hospital of China Medical University，Shenyang 110004, China | 41 | 11 | 752-755 | 10.3760/cma.j.issn.0529-5807.2012.11.009 |
| 6 | Zhang Xuedong, Chen Shichao, Yang Haizhen, 2012 | Bilateral testicular spermatocytic seminoma: A case report and literature review | Journal of Clinical and Pathological Research | Zhang Xuedong | Zhang Xuedong, Chen Shichao and Yang Haizhen. | Department of Pathology, Liaocheng People's Hospital, Shandong Province, Liaocheng 252000, China | 28 | 05 | 583-585 | 10.3969/j.issn.1001-7399.2012.05.031 |
| 7 | Shao Mingming, Zhang Xiaojun, Jin Meishan, Zhang Haitao, Xu Ning, 2012 | Bilateral testicular spermatocytic seminoma: A case report and literature review | National Journal of Andrology | Shao Mingming | Shao Mingming, Zhang Xiaojun, Jin Meishan, Zhang Haitao and Xu Ning | Department of Urology, The First Hospital of Jilin University, Changchun, Jilin 130021, China | 18 | 11 | 1036-1038 | 10.13263/j.cnki.nja.2012.11.017 |
| 8 | Wang Xiang, Feng Yongheng, Liu Pengtao, 2011 | One case: spermatocytal seminoma of testis | Journal of Practical Radiology | Wang Xiang | Wang Xiang, Feng Yongheng and Liu Pengtao | Armed Police Shaanxi Corps Hospital, Shaanxi, Xi'an 710054, China | 27 | 10 | 1606-1606 | 10.3969/j.issn.1002-1671.2011.10.049 |
| 9 | Gao Likun, Wang Xiaomei, Chen Zhuohuai, 2009 | A case of seminoma with sarcomatous components in testicular spermatogonia and a review of the literature | Compilation of papers from the 2009 Annual Academic Conference of the Pathology Branch of Chinese Medical Association | Gao Likun | Gao Likun, Wang Xiaomei and Chen Zhuohuai | Department of Pathology, Shenzhen People's Hospital, China | NA | NA | 132-134 | NA |
| 10 | Jia Yong, Guo Lina, Liu Tonghua, 2004 | A case of testicular spermatocytic seminoma combined with carcinosarcoma | Chinese Journal of Pathology | Jia Yong | Jia Yong, Guo Lina and Liu Tonghua. | Chinese Academy of Medical Sciences, 100730, China | 33 | 4 | 391-392 | 10.3760/j.issn:0529-5807.2004.04.033 |
| 11 | Du Jiangping, Lin Chao, Shi Shaoxian, Liu Xiaoyan, Li Haitao, 2003 | A case report of bilateral testicular spermatocytic seminoma | Journal of Modern Urology | Du Jiangping | Du Jiangping, Lin Chao, Shi Shaoxian, Liu Xiaoyan and Li Haitao | Department of Urology, Fengfeng Mining Bureau General Hospital, Handan, Hebei, China | 8 | 03 | 170-170 | NA |
| 12 | Li Zhi, Yun Qiu, Liu Weiguo, 2002 | A case of testicular spermatocytic seminoma | Journal of Clinical Radiology | Li Zhi | Li Zhi, Yun Qiu and Liu Weiguo | Department of Radiology, Armed Police Guangdong Corps Hospital, Guangzhou, China | 21 | 08 | 595-595 | 10.3969/j.issn.1001-9324.2002.08.023 |
| 13 | Zhang Kai, Kong Xiangtian, Yang yong, Zhang Xiaochun, Wu Shiliang, Xia Tongli et al., 1998 | A case of spermatocytic seminoma | Chinese Journal of Surgery | Zhang Kai | Zhang Kai, Kong Xiangtian, Yang yong, Zhang Xiaochun, Wu Shiliang, Xia Tongli and Xue Zhaoying. | Institute of Urology, Peking University First Hospital, 100034, China | 36 | 07 | 432-432 | NA |
| 14 | Yan Xiangjin, Ou Yangxue, Hu Aixia, 1995 | A case of testicular spermatocytic seminoma | Chinese Journal of Pathology | Yan Xiangjin | Yan Xiangjin, Ou Yangxue and Hu Aixia | Department of Pathology, PLA 85 Hospital, China | 24 | 05 | 287-287 | NA |
